# Sorbic acid triggers rapid formation of proteasome storage granules in *Saccharomyces cerevisiae*

**DOI:** 10.15698/mic2026.08.886

**Published:** 2026-08-12

**Authors:** Shoei Tanaka, Mitsuki Imajo, Mieko Hayashi, Akira Matsuura, Shingo Izawa

**Affiliations:** 1 Department of Applied Biology, Graduate School of Science and Technology, Kyoto Institute of Technology, Sakyo-ku, Kyoto 606-8585, Japan; 2 Department of Biology, Graduate School of Science, Chiba University, Chiba 263-8522, Japan

**Keywords:** sorbic acid, acidification, proteasome, proteasome storage granules, proteolysis, UBA-UBL shuttle factors, *Saccharomyces cerevisiae*

## Abstract

Proteasomes are primarily located within the nuclei of proliferating cells; however, their localization changes dynamically in response to environmental conditions. *Saccharomyces cerevisiae* forms proteasome storage granules (PSGs) in the cytoplasm upon glucose depletion, mitochondrial stress, transition to quiescence, or acetic acid stress, with intracellular acidification acting as a key trigger. Despite possessing similar dissociation constants, sorbic acid and acetic acid are noted to exert different physiological effects on yeast cells. In this study, we demonstrate that sorbic acid induces the fastest PSG formation ever reported (within 30 min) at lower concentrations than acetic acid, accompanied by the inhibition of proteasomal proteolysis. Conversely, our analysis of the required factors revealed that, similar to acetic acid, the proteasome subunits Sem1 and Rpn13 are essential for sorbic acid-induced PSG formation. In contrast, the shuttle factors Dsk2 and Rad23, along with the E3 ubiquitin ligase Hul5—which are required for PSG formation under mitochondrial stress or quiescence—are dispensable for sorbic acid-induced PSG formation. These findings not only identify a novel condition for rapid PSG formation but also provide new insights into the physiological effects of sorbic acid on the proteasome, highlighting both its similarities to and differences from acetic acid.

## INTRODUCTION

The 26S proteasome is a highly conserved proteolytic machinery present in all eukaryotes, playing a pivotal role in maintaining proteostasis (protein homeostasis) and regulating diverse cellular functions 1, 2. It mediates the degradation of damaged or misfolded proteins and regulatory proteins (e.g., cyclins, transcription factors) mainly via ubiquitination; this pathway is known as the ubiquitin-proteasome system (UPS) 2–4. The 26S proteasome is a massive ATP-dependent protease complex (approximately 2.5 MDa), comprising a 20S core particle (20S CP) and one or two 19S regulatory particles (19S RPs) 5–7. The 20S CP is a cylindrical structure composed of four stacked heptameric rings (two outer α -rings and two inner β -rings), where substrates are degraded within the internal lumen by trypsin-like, chymotrypsin-like, and caspase-like activities 3, 4, 8, 9. The 19S RP, comprising 19 distinct subunits, caps one or both ends of the 20S CP. It utilizes ATP to mediate the recognition of ubiquitin chains, substrate deubiquitination, protein unfolding, and the subsequent translocation of substrates into the 20S CP 3, 4, 8, 9. Beyond the ATP-dependent pathway, the free 20S CP can degrade proteins in a ubiquitin- and ATP-independent manner. Specifically, proteins with unstructured regions due to oxidative stress are highly susceptible to degradation by the free 20S CP 10, 11.

**Figure 1:**
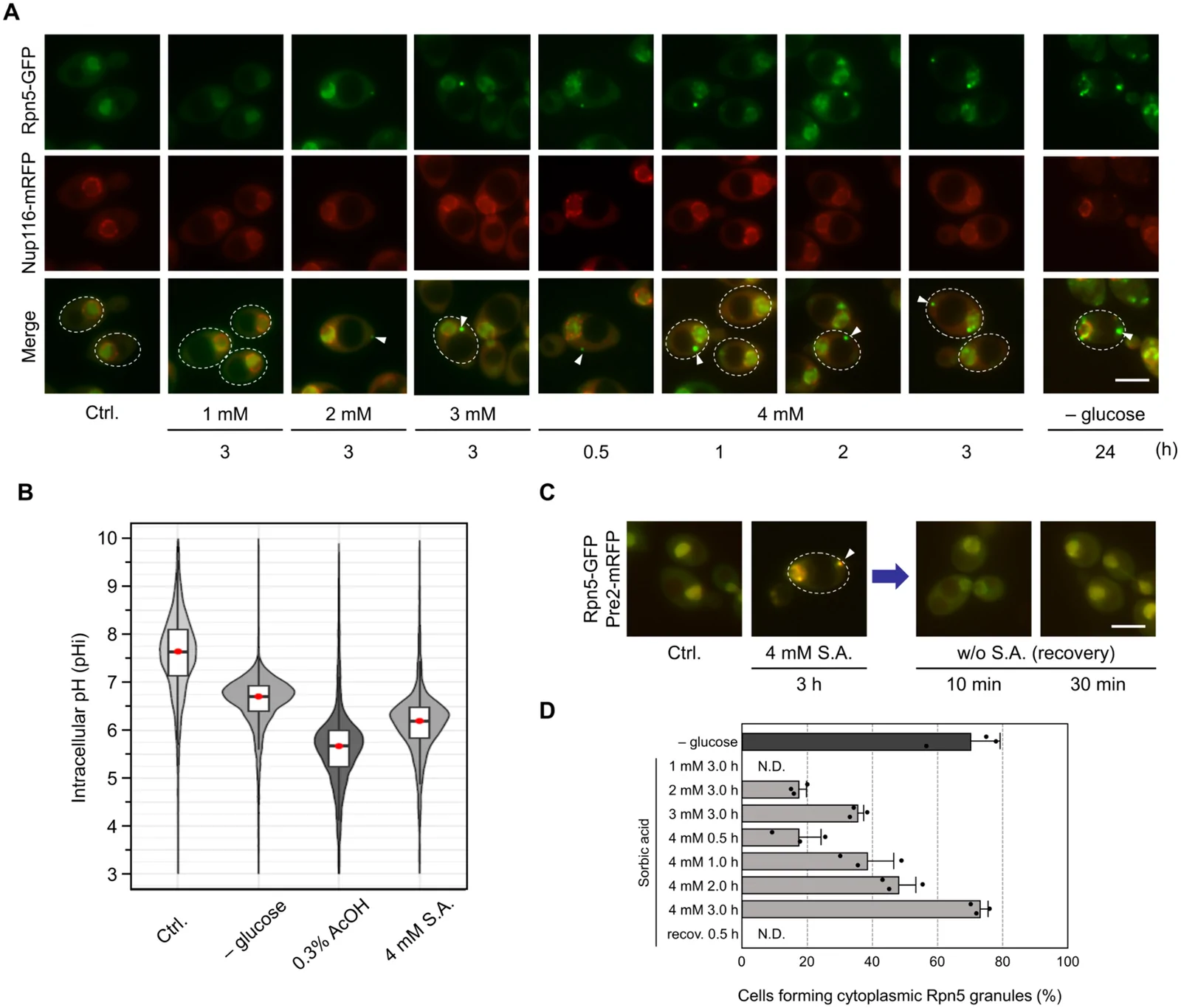
Sorbic acid stress causes the formation of cytoplasmic proteasome granules in yeast cells. **(A)** Yeast cells expressing Rpn5-GFP (a 19S RP lid subunit) and Nup116-mRFP (a nuclear envelope marker) were treated with sorbic acid stress or glucose depletion for the indicated times. White arrowheads denote cytoplasmic Rpn5 granules. **(B)** Cells were subjected to stress conditions: glucose depletion, 0.3% acetic acid, or 4 mM sorbic acid for 1 h. Subsequently, pHi was determined by measuring pHluorin2 via flow cytometry. **(C)** Reversibility of sorbic acid-induced proteasome granules. Yeast cells treated with 4 mM sorbic acid for 3 h were transferred to fresh SD medium without sorbic acid. **(D)** Quantitative analysis of cells containing cytoplasmic proteasome granules. Data are based on three independent experiments, with a total of over 300 living cells observed per condition. No granule formation was observed under non-stressed conditions. Scale bar, 5 μ m. S.A., sorbic acid.

The 26S proteasome is primarily located in the nucleus of proliferating cells; however, its localization is dynamically regulated depending on stress and growth conditions 12–15. Stress conditions, including amino acid starvation, hyperosmotic shock, and cellular senescence, induce the formation of nuclear proteasome condensates in mammalian cells 14–18. These condensates sequester ubiquitinated proteins and the proteasome shuttle factor RAD23B, which contains ubiquitin-associated (UBA) and ubiquitin-like (UBL) domains 14, 17, 19–21. Yeast proteasomes also localize to the perinuclear region in quiescent cells and in cells treated with the proteasome inhibitor MG132 22, 23. In the cytoplasm, the formation of proteasome storage granules (PSGs) is well known as a yeast-specific phenotype 18. PSGs are also known to contain the UBA-UBL shuttle factors Dsk2 and Rad23 (a yeast homolog of mammalian RAD23B), as well as ubiquitinated proteins 24, 25. A model has been proposed in which the formation of nuclear proteasome condensates and PSGs is driven by liquid–liquid phase separation (LLPS), mediated by multivalent interactions between ubiquitinated proteins, shuttle factors, and proteasomes 12, 14, 24, 26. In contrast, a recent study has shown that PSGs are paracrystalline arrays composed of bundled fibers formed by the stacking of proteasome trimers 27. While the mechanisms underlying PSG formation are thus becoming increasingly elucidated, their physiological significance remains largely elusive.

PSG formation is induced by acute glucose depletion, quiescence, and mitochondrial stress, all of which reduce intracellular pH (pHi) and ATP levels, alongside altering metabolism 12, 18, 24, 26–30. Furthermore, it has been demonstrated that intracellular acidification alone is sufficient to induce PSG formation 26. We recently reported that sublethal acetic acid stress (52.5 mM), which also reduces pHi 31–33, induces PSG formation with faster kinetics than conventional methods such as acute glucose depletion 25. We also confirmed that the factors required for PSG formation differ among these induction conditions.

Sorbic acid is a lipophilic weak acid that has a dissociation constant similar to that of acetic acid (p*K*a = 4.76 at 25 ${}^{\circ }$ C). Interestingly, while sorbic acid exhibits fungistatic effects on *S. cerevisiae* at significantly lower concentrations than acetic acid, it exerts a more modest impact on pHi reduction 34–38. Although it has been suggested that sorbic acid and acetic acid differ in their effects on yeast and bacterial cells, as well as in the corresponding yeast responses 39–42, the precise effects of sorbic acid on yeast cells are not yet fully understood. Similarly, the specific responses of yeast to this stressor remain insufficiently characterized.

Based on this background, we investigated whether sorbic acid possesses the capacity to induce PSG formation and compared its efficacy with that of acetic acid. Our results show that 4 mM sorbic acid rapidly induces PSG formation, exceeding the efficacy of acetic acid and other known PSG induction conditions. Additionally, we found that sorbic acid treatment inhibits proteasomal proteolysis. To elucidate the underlying mechanisms of PSG formation, we compared the factors required for PSG formation between sorbic acid and other established induction conditions. Our results offer novel insights into the physiological response of yeast to sorbic acid and the regulatory pathways of PSG formation, identifying similarities and differences in the regulatory pathways of PSG formation induced by acetic acid.

## RESULTS

### Sorbic acid causes the formation of PSGs

To investigate whether sorbic acid induces PSG formation, we used Rpn5-GFP as a canonical PSG marker 24, 26, 43. Following a previous report that sublethal concentrations of acetic acid trigger PSG formation 25, we evaluated sorbic acid at concentrations up to 4 mM, a range established as a sublethal in prior studies 44, 45. While 1 mM sorbic acid failed to induce cytoplasmic Rpn5 granules, concentrations of 2 mM or higher successfully triggered their formation (Figure 1**A**). Specifically, 4 mM sorbic acid induced the formation of cytoplasmic Rpn5 granules in approximately 20% of the cell population within 30 min, increasing to over 70% after 3 h (Figures 1**A** and 1**D**). In contrast, concentrations of 3 mM or lower resulted in Rpn5 granule formation in less than half of the cells after 3 h. Based on these results, 4 mM sorbic acid was used for all subsequent experiments. Treatment with 4 mM sorbic acid was confirmed to reduce pHi (Figure 1**B**).

Upon removal of sorbic acid by medium exchange, the cytoplasmic Rpn5-GFP granules induced by 4 mM sorbic acid treatment rapidly dissociated, and the signal relocalized to the nucleus (Figure 1**C**). This reversibility supports the notion that sorbic acid-induced Rpn5 granules are characteristically similar to PSGs 24, 25.

Next, we examined the localization changes of other proteasome subunits. Similar to Rpn5, other subunits formed cytoplasmic granules with nearly identical granule formation rates under 4 mM sorbic acid stress (Figure 2). Given that all examined proteasome subunits formed cytoplasmic granules and co-localized with Pre2 or Pre4 (20S CP subunits), we conclude that sorbic acid induces the formation of PSGs, mirroring the effects of acetic acid.

**Figure 2:**
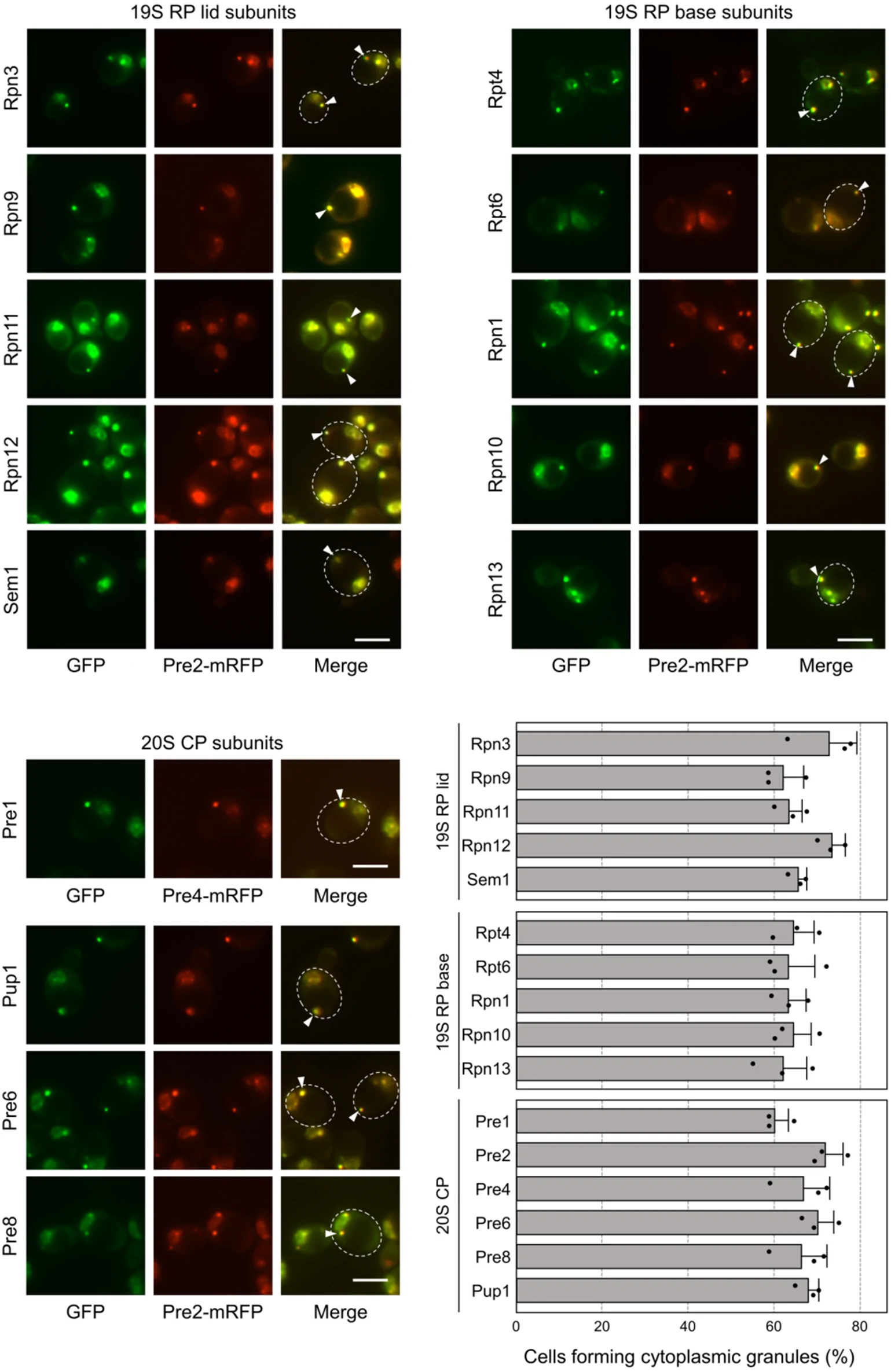
19S RP subunits form granules with 20S CP subunits under sorbic acid stress. Cells expressing one of the GFP-tagged proteasome subunits and either mRFP-tagged Pre2 ( β 5 of 20S CP) or Pre4 ( β 7 of 20S CP) were treated with 4 mM sorbic acid for 3 h. White arrowheads indicate the cytoplasmic proteasome granules. Scale bar, 5 μ m. The right bottom panels indicate the granule formation rate of each proteasome subunit examined in this study. No granule formation was observed in any subunit under non-stressed conditions.

### Sorbic acid inhibits proteasomal proteolysis and causes the accumulation of ubiquitinated proteins

Next, we examined the relationship between PSG formation and proteasome activity under sorbic acid stress. *In vivo* proteasomal proteolysis was monitored via cycloheximide (CHX)-chase analysis using an auxin-inducible degron (AID) system 46. Although the addition of indole-3-acetic acid (IAA) rapidly induced the proteasomal degradation of Paf1–AID^*^–6FLAG under non-stressed conditions 47, 48, this effect was clearly suppressed by 4 mM sorbic acid (Figures 3**A** and 3**C**).

We also examined the effect of sorbic acid on ubiquitin-independent proteasomal proteolysis by monitoring the degradation of ornithine decarboxylase Spe1, which is known to be degraded in a ubiquitin-independent manner in the presence of polyamines such as spermidine 48–51. CHX-chase analysis revealed that spermidine-induced proteasomal proteolysis of Spe1-3HA was also inhibited in the presence of 4 mM sorbic acid (Figures 3**B** and 3**C**). The extent of this inhibition was dependent on the sorbic acid concentration (1–4 mM) and showed a trend toward correlation with the degree of PSG formation (Figure S1).

**Figure 3:**
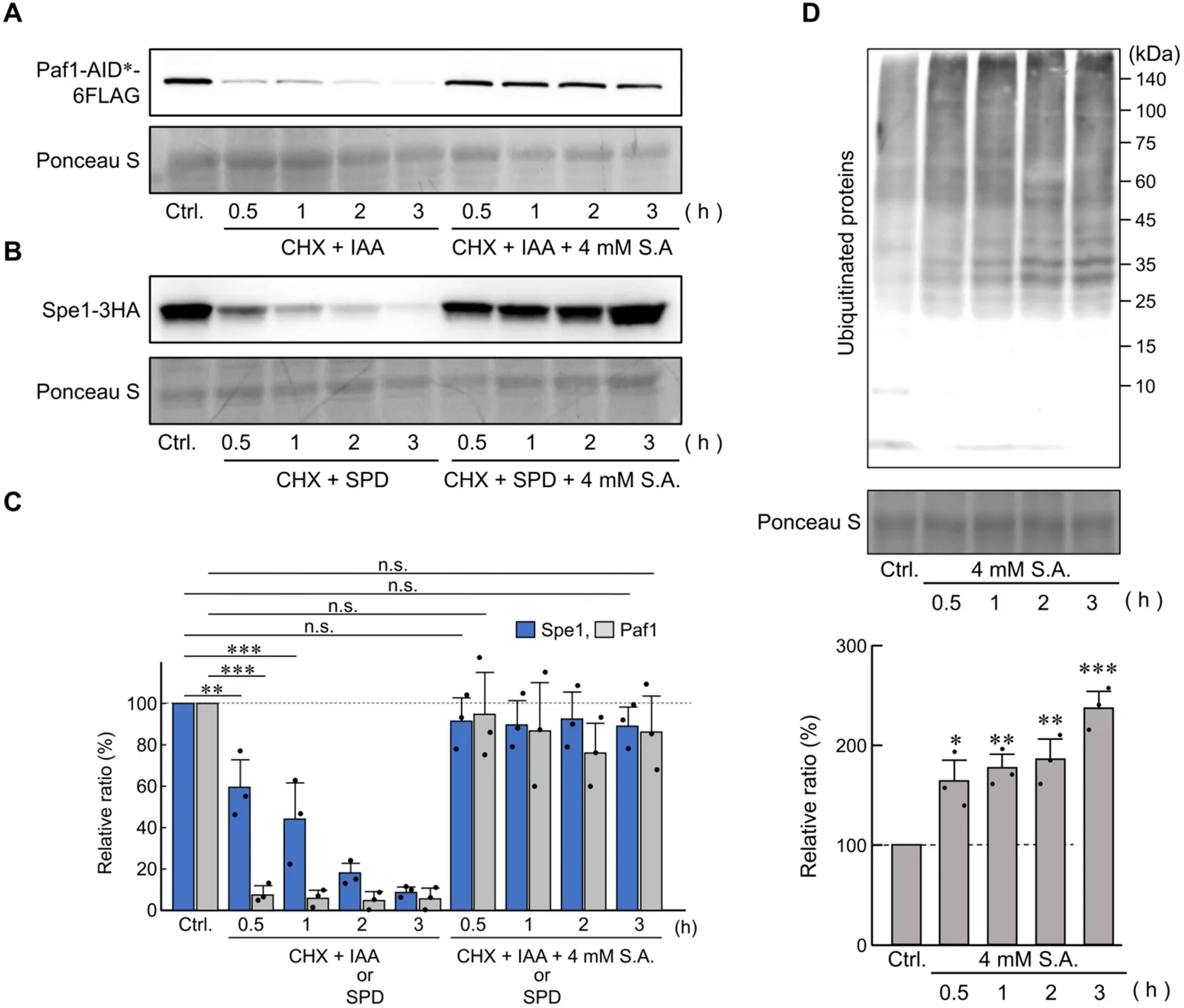
Sorbic acid stress inhibits proteasomal proteolysis. **(A)** Assessment of proteasomal proteolysis efficiency using the auxin-inducible degron (AID) system. Cells expressing Paf1–AID^*^–6FLAG were treated with 0.75 mM indole-3-acetic acid (IAA) and 200 μ g/mL cycloheximide (CHX), with or without 4 mM sorbic acid, for the indicated durations. **(B)** Assessment of ubiquitin-independent proteasomal degradation using Spe1-3HA. Cells were treated with 1 mM spermidine (SPD) and 200 μ g/mL CHX to induce Spe1 degradation, in the presence or absence of 4 mM sorbic acid. **(C)** Quantitative analysis of Paf1–AID*–6FLAG and Spe1-3HA levels normalized to Ponceau S staining. n.s., statistically not significant; ${}^{**}$ *p* < 0.01, ${}^{***}$ *p* < 0.005 (Dunnett’s test, *n* = 3). **(D)** The levels of ubiquitinated proteins were detected by western blotting using an anti-ubiquitin antibody. Ubiquitinated protein levels were normalized to Ponceau S staining (*right bottom panel*). Statistical significance was assessed using Dunnett’s test (*n* = 3); ${}^{*}$ *p* < 0.05, ${}^{**}$ *p* < 0.01, ${}^{***}$ *p* < 0.005. S.A., sorbic acid.

Furthermore, increased levels of ubiquitinated proteins were observed in yeast cells under sorbic acid stress (Figure 3**D**). These results clearly indicate that proteasomal proteolysis was inhibited under sorbic acid stress, similar to the effect of acetic acid stress 25.

### Shuttle factors are dispensable for PSG formation under sorbic acid stress

The UBA–UBL shuttle factors, Dsk2 and Rad23, are crucial for PSG formation during mitochondrial stress or quiescence, but are dispensable for that induced by acute glucose depletion or acetic acid 24, 25. To determine their role in response to sorbic acid stress, we monitored PSG formation in *dsk2* Δ *rad23* Δ cells. Exposure to 4 mM sorbic acid induced PSG formation in over 50% of the double-mutant cells (Figure 4**A**), indicating that Dsk2 and Rad23 are not strictly required for PSG formation under sorbic acid stress.

Notably, however, both Dsk2-GFP and Rad23-GFP co-localized with the proteasome subunit Pre2-mRFP upon sorbic acid stress (Figure 4**B**). These results suggest that while these shuttle factors are sequestered into PSGs under this condition—consistent with other induction conditions 24, 25—they are not the primary drivers of PSG formation in this context.

**Figure 4:**
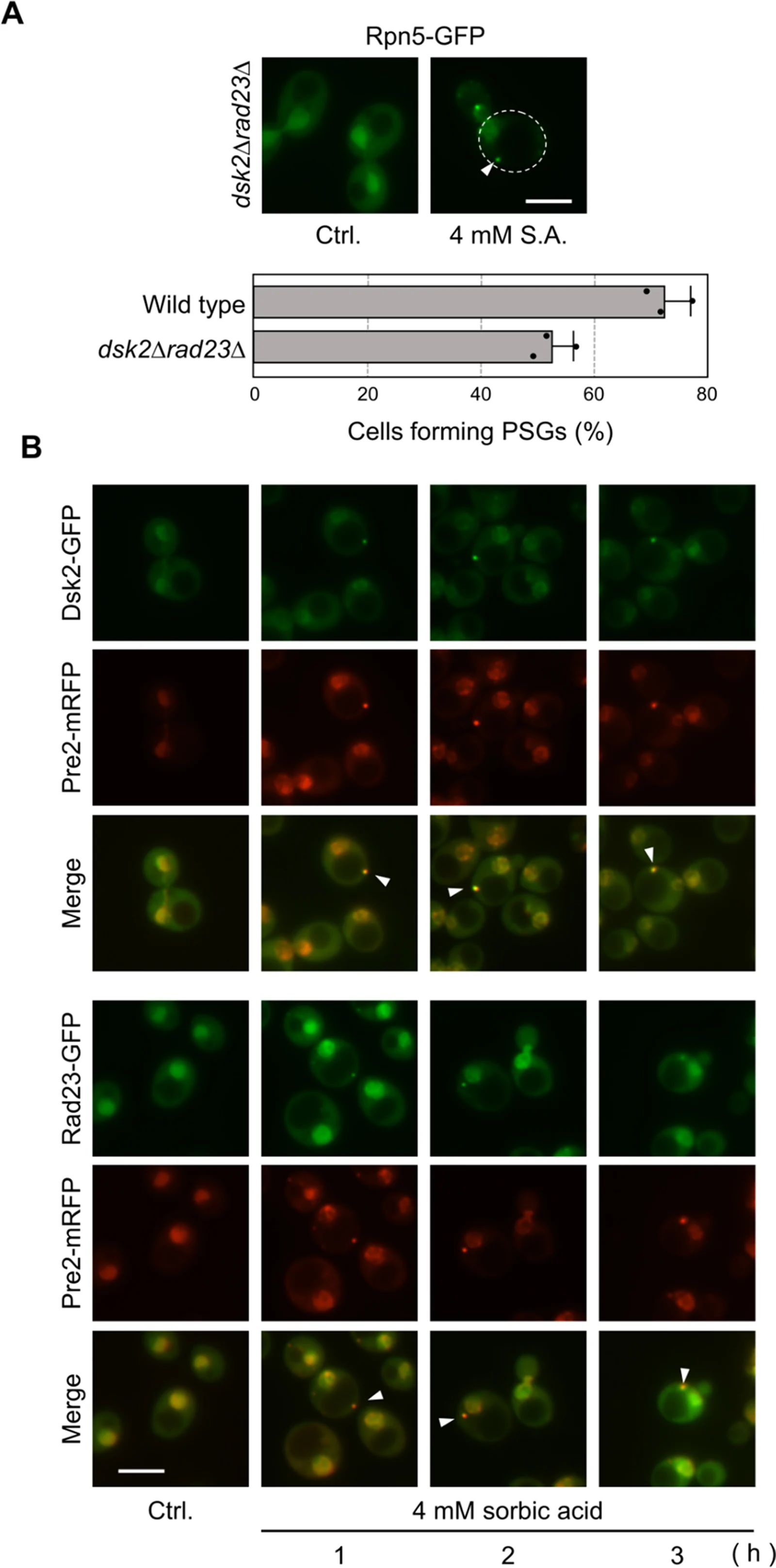
Dsk2 and Rad23 colocalize with PSGs but are not essential for PSG formation under sorbic acid stress. (**A**) PSG formation was examined using Rpn5-GFP in *dsk2* Δ *rad23* Δ cells under 4 mM sorbic acid stress for 3 h. White arrowheads indicate PSGs. The lower panel shows the PSG formation rates of wild-type and *dsk2* Δ *rad23* Δ cells exposed to 4 mM sorbic acid for 3 h. **(B)** Intracellular localization of Dsk2-GFP and Rad23-GFP was examined in wild-type cells expressing Pre2-mRFP. Cells were exposed to 4 mM sorbic acid stress for the indicated periods. White arrowheads indicate granules where Dsk2-GFP or Rad23-GFP colocalizes with Pre2-mRFP. Scale bar, 5 μ m.

Another yeast UBA–UBL shuttle factor, Ddi1, possesses a viral protease domain that cleaves substrates with long polyubiquitin chains 24, 52. Consistent with this, *ddi1* Δ cells exhibit accumulation of polyubiquitinated substrates and spontaneous PSG formation even under non-stressed conditions 24, 51. While Ddi1 is known to form cytoplasmic condensates in quiescent cells and under glucose depletion or acetic acid stress, these condensates localize independently of PSGs 24, 25. We found that sorbic acid also induced the formation of Ddi1 condensates, which localized independently of PSGs (Figure 5).

**Figure 5:**
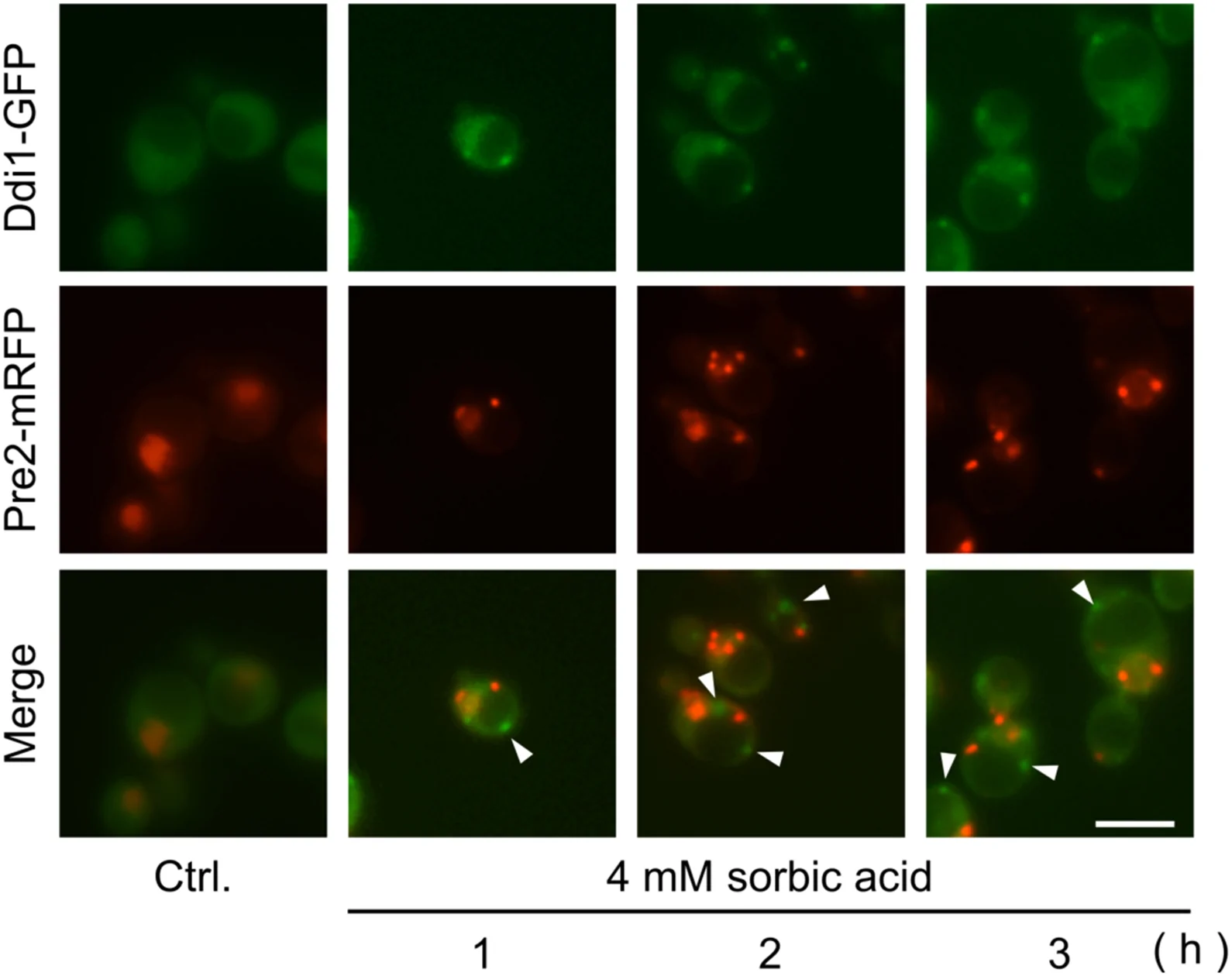
Sorbic acid induces Ddi1 condensate formation. Ddi1-GFP localization was examined in wild-type yeast cells expressing Pre2-mRFP. Cells were subjected to sorbic acid stress for the indicated periods. White arrowheads indicate Ddi1-GFP condensates that did not colocalize with Pre2-mRFP. Scale bar, 5 μ m.

### Sem1 and Rpn13 are indispensable for sorbic acid-induced PSG formation

In addition to UBA-UBL shuttle factors, the factors required for PSG formation vary depending on the induction conditions 23–25, 53. In this study, we examined sorbic acid-induced PSG formation in strains deficient in the *HUL5* gene or non-essential proteasome subunit genes (*PRE9*, *RPN10*, *RPN13*, and *SEM1*).

Sem1, which is indispensable across all previously reported induction conditions 25, 54, was also essential for sorbic acid-induced PSG formation; accordingly, *sem1* Δ cells failed to form PSGs in response to sorbic acid stress (Figure 6). Similarly, Rpn13—required for PSG formation under acetic acid stress but not during glucose depletion 24, 25—was necessary for sorbic acid-induced PSG formation. The essentiality of Sem1 and Rpn13 was further verified through complementation assays employing pRS-*SEM1* and pRS-*RPN13* (Figure 6). In contrast, the deletion of *PRE9* and *RPN10* had no discernible effect on PSG formation under sorbic acid stress.

Hul5, a proteasome-interacting E3 ubiquitin ligase, is required for PSG formation during mitochondrial stress or quiescence, but is dispensable for PSG formation triggered by acute glucose depletion or acetic acid stress 23, 25. Similar to observations under glucose depletion and acetic acid stress, *hul5* Δ cells induced PSG formation under sorbic acid stress (Figure 6). These results indicate that there is no difference between sorbic acid and acetic acid regarding the factors required for PSG formation, as far as we investigated in this study. The survival rates of the null mutants used in this study remained high after treatment with 4 mM sorbic acid for 3 h (Figure 7).

**Figure 6:**
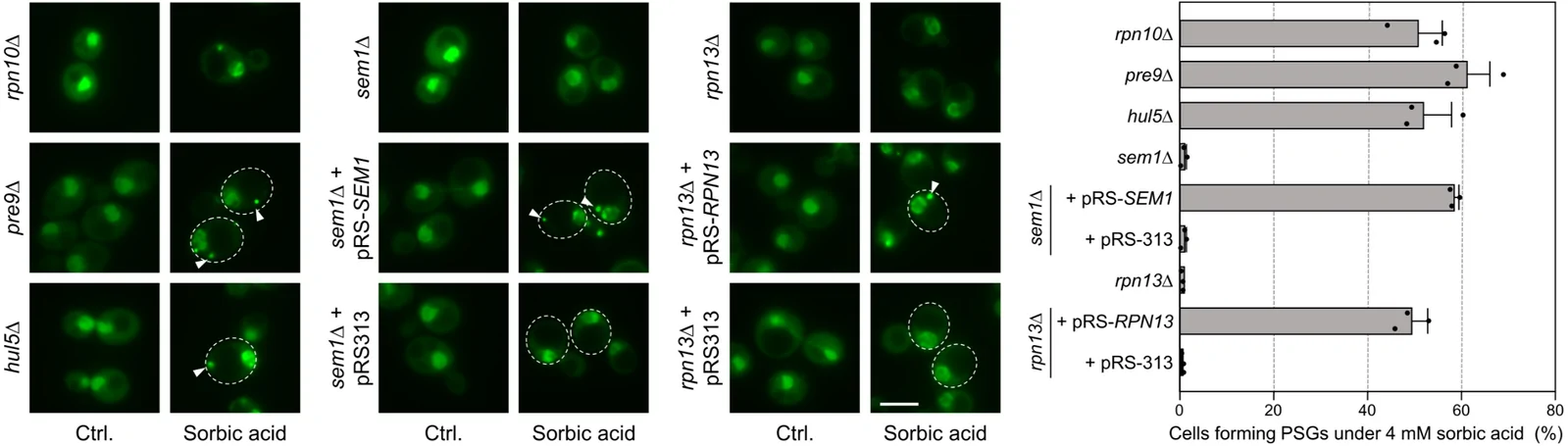
PSG formation is mitigated in *rpn13* Δ and *sem1* Δ cells under sorbic acid stress. PSG formation in *rpn10* Δ , *pre9* Δ , *hul5* Δ , *sem1* Δ , and *rpn13* Δ cells was examined using Rpn5-GFP. Cells were treated with 4 mM sorbic acid for 3 h. *sem1* Δ and *rpn13* Δ cells were transformed with pRS313-*SEM1* and pRS313-*RPN13*, respectively, while the empty vector (pRS313) served as a control. White arrowheads indicate PSGs and the PSG formation rates after 3 h of treatment with 4 mM sorbic acid are shown in the right panel. No granule formation was observed under non-stressed conditions. Scale bar, 5 μ m.

**Figure 7:**
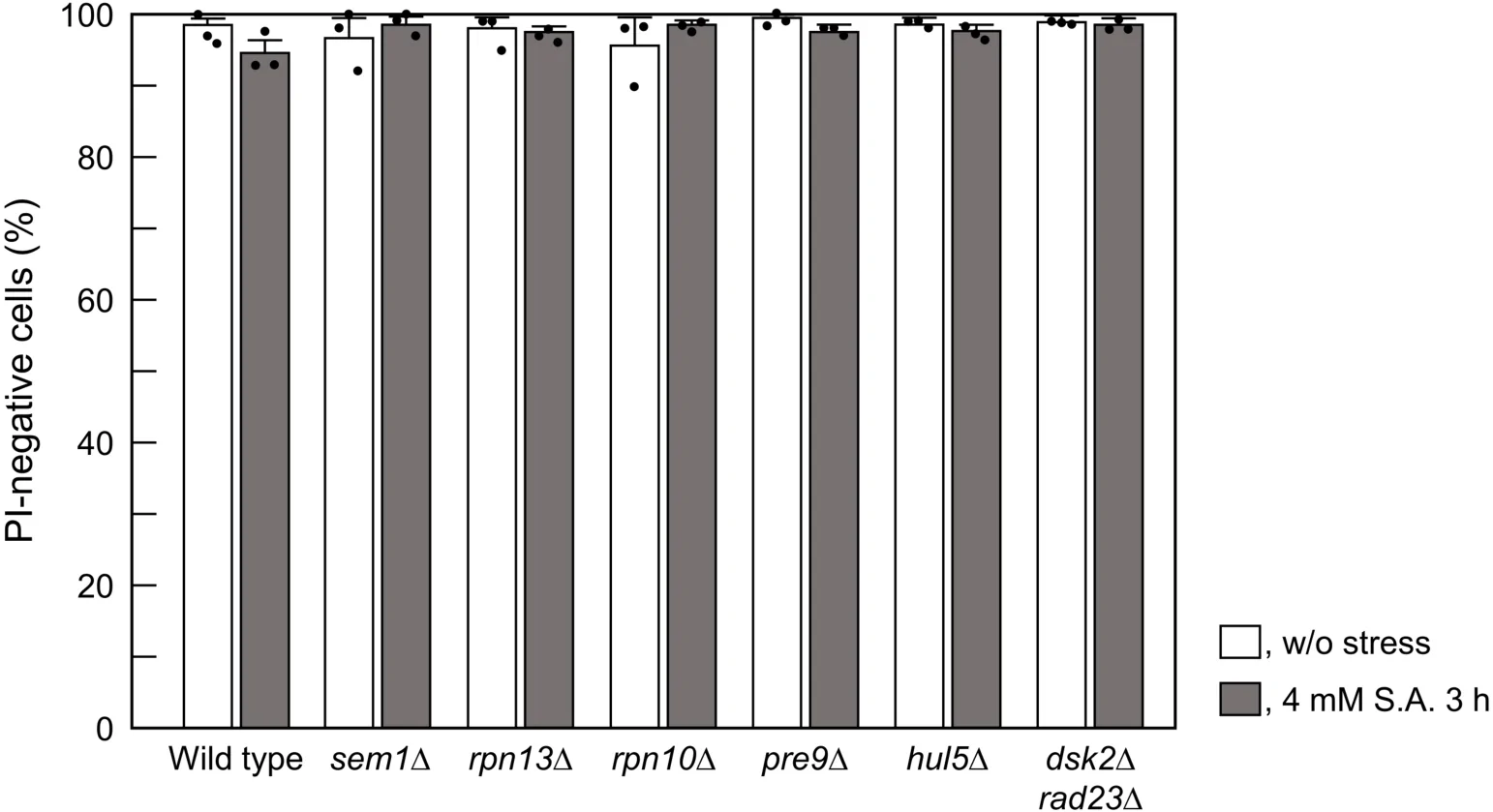
Cell viability following sorbic acid stress treatment. Cells were exposed to 4 mM sorbic acid in SD medium for 3 h. Cell viability was then assessed by propidium iodide (PI) staining, with PI-negative cells considered viable. Data are based on three independent experiments, with a total of over 300 living cells observed per condition. S.A., sorbic acid.

## DISCUSSION

This study revealed a novel physiological effect of sorbic acid on yeast proteasomes. Treatment with 4 mM sorbic acid induced the formation of PSGs, accompanied by the inhibition of proteasomal proteolysis. The most noteworthy finding is that sorbic acid triggers PSG formation with remarkably accelerated kinetics. Compared to conventional induction conditions—such as glucose deprivation, transition to quiescence, or mitochondrial inhibition, which require 12 to 24 h for sufficient PSG formation 12, 24, 29 or acetic acid stress, which necessitates 9 h 25—4 mM sorbic acid induced detectable PSG formation within approximately 30 min. Furthermore, 4 mM sorbic acid stress induced PSGs in the majority of the cell population within 3 h. This rapid induction provides a significant methodological advantage for enhancing the throughput and efficiency of PSG research.

Consistent with its established antimicrobial properties 37, 39, 41, sorbic acid induced PSG formation at lower concentrations than acetic acid 25. In contrast, previous studies have reported that sorbic acid exerts a weaker effect on pHi reduction than acetic acid 36, 37. Our data corroborate these reports, demonstrating that sorbic acid-induced pHi reduction was more modest than that caused by acetic acid, even at PSG-inducing concentrations. Nevertheless, the degree of acidification under sorbic acid stress exceeded that triggered by glucose depletion. These results suggest that, unlike lactic acid and 10% ethanol 25, sorbic acid is capable of reducing pHi below the critical threshold required for PSG formation 26. Notably, the kinetics of PSG formation did not correlate with the magnitude of the pHi decline. Although the degree of acidification varied depending on the stressors 26, 30, 33, 55, these differences were not reflected in the timeline of PSG formation. This suggests that the extent of acidification is not the rate-limiting factor governing the kinetics of PSG assembly. Furthermore, the distinct kinetics of PSG formation between acetic and sorbic acids may be influenced by modes of action independent of their pHi-lowering effects 41, 45, 56–58.

PSG formation induced by the transition to quiescence or mitochondrial stress is significantly impaired in *dsk2* Δ *rad23* Δ and *hul5* Δ cells 23–25, 53. In contrast, these genetic deficiencies have a negligible impact on PSG formation triggered by sorbic acid (Figures 4A and 6) or acetic acid stress 25. While the requirements for Rpn10 and Rpn13 in PSG formation vary depending on the induction conditions 24, 25, Rpn10 was dispensable for sorbic acid-induced PSG formation, consistent with its role in the response to acetic acid stress 25. Conversely, Rpn13 was essential for PSG formation under both sorbic and acetic acid stress conditions. Additionally, we confirmed that Sem1 is essential for PSG formation under all conditions examined 25, 54. Despite differences in formation kinetics and effective concentrations, the shared necessity of these factors suggests a highly similar underlying mechanism for PSG formation induced by sorbic and acetic acids. Elucidating the effects of various weak acids may provide critical insights into the physicochemical triggers and the diversity of induction pathways of PSG formation.

Although both Rpn10 and Rpn13 serve as ubiquitin receptors for the proteasome, only Rpn13 is essential for sorbic acid-induced PSG formation. Although further studies are warranted, as discussed previously 25, this difference may tentatively be explained by distinct post-translational modifications of these two subunits. In contrast, Sem1 is required for PSG formation under all tested conditions, acting as a chaperone for 19S RP assembly 59. Previous studies have also demonstrated that *sem1* deletion reduces 26S proteasome stability and impairs its activity 60, 61. Together, these findings suggest that the inefficient 19S RP assembly in *sem1* Δ cells limits the cellular abundance of fully assembled 26S proteasomes, consequently impairing PSG formation.

The present study also demonstrates that sorbic acid inhibits proteasomal proteolysis and causes the accumulation of ubiquitinated proteins, paralleling the effects of acetic acid stress and other PSG induction conditions 25, 62–66. Combined with our recent finding that sorbic acid suppresses translation activity 45, it is clear that sorbic acid inhibits both protein synthesis and degradation within the yeast proteostasis network. Given that all known PSG induction conditions—including sorbic acid stress—lead to decreased intracellular ATP levels and reduced ATP consumption 24, 27, 42, 67–70, this suppression likely represents an energy-saving response necessitated by limited ATP availability. Under glucose starvation, yeast cells enter an energy-saving and protective survival state 71; a comparable state may be induced by sorbic acid stress. Furthermore, since ATP depletion destabilizes the proteasome 15, 62, 65, our results suggest that the inhibition of proteasomal proteolysis is both a consequence and a driver of the metabolic stress response, forming a feedback loop that regulates energy status and proteostasis.

It is also of considerable interest to compare sorbic acid-induced PSGs with mammalian LLPS-driven proteasome condensates 14. While mammalian nuclear proteasome condensates facilitate the degradation of unassembled ribosomal proteins 14, specific substrates for proteasomes that co-localize with yeast PSGs remain to be identified. Furthermore, yeast proteasomal activity is significantly impaired under stress conditions 25, including sorbic acid treatment. Collectively, these observations suggest that yeast PSGs likely serve a distinct physiological role rather than acting as proteolytic hubs.

A recent cryo-electron tomography study demonstrated that quiescent phase-induced PSGs form paracrystalline arrays in an inactive S1-like conformation 27. Because the biophysical properties and ultrastructure of sorbic acid-induced PSGs were not investigated in the present study, whether these granules also adopt a paracrystalline structure or represent a distinct type of proteasome condensate remains to be elucidated. Further structural analyses utilizing cryo-electron tomography will be indispensable to define their precise organization. In line with this, the nuclear localization pattern of proteasomes under sorbic acid stress appears to differ from that observed during the quiescent phase 25. This finding may reflect distinct underlying mechanisms or kinetics of PSG formation associated with these two stressors.

In conclusion, this study demonstrates that sorbic acid induces rapid PSG formation and suppresses proteasomal proteolysis. This proteostatic disruption likely contributes to its fungistatic effects by interfering with the precise regulation of key proteins, such as cyclins. Furthermore, sorbic acid represents a valuable tool for promptly inducing PSG formation. This rapid induction—occurring within a few hours—significantly enhances downstream experimental efficiency, particularly when screening for factors required for PSG formation, while minimizing the confounding effects typically associated with prolonged stress treatments.

## MATERIALS AND METHODS

### Yeast strains and stress treatment

The wild-type strain BY4742 (*MAT* α *ura3* Δ *0 his3* Δ *1 leu2* Δ *0 lys2* Δ *0*) and its isogenic knockout mutants (*hul5* Δ *::kanMX*, *pre9* Δ *::kanMX*, *rpn10* Δ *::kanMX*, *rpn13* Δ *::kanMX*, and *sem1* Δ *::kanMX*) were purchased from Open Biosystems (Huntsville). Yeast strains expressing GFP- or mRFP-tagged proteasome subunits and *dsk2* Δ *rad23* Δ used in this study are as described in our recent publication 25. Rpn5-GFP is integrated into the *RPN5* locus on the genome (present at a single copy per cell) and is under the expression control of the endogenous *RPN5* promoter. All other proteasome subunits tagged with GFP or mRFP were similarly integrated into the genome. Synthetic defined (SD) medium (2% glucose, 0.67% yeast nitrogen base without amino acids, 20 mg/L uracil, 30 mg/L L-lysine HCl, 100 mg/L L-leucine, and 20 mg/L L-histidine HCl, pH 5.6) was used for cultivation of yeast cells with reciprocal shaking (120 rpm) at 28 ${}^{\circ }$ C, and exponentially growing cells were harvested at an optical density at 600 nm (OD ${}_{600}$ ) of 0.5. The sorbic acid stress treatment was conducted by resuspending the harvested cells in fresh SD medium containing sorbic acid. The cell death rate was assessed using propidium iodide (PI) staining 72.

### CHX-chase analysis and western blotting

The efficiency of proteasomal degradation was monitored by CHX-chase analysis using 200 μ g/mL CHX (Nacalai Tesque, Kyoto, Japan) 48. Ubiquitinated proteins were detected using an anti-ubiquitin antibody (P4D1; Santa Cruz Biotechnology, Dallas, TX, USA). Other antibodies used for western blotting included anti-FLAG antibody (F1804; Sigma-Aldrich, St Louis, MO, USA), anti-HA monoclonal antibody (M180–3; Medical and Biological Laboratories Co., Ltd., Tokyo, Japan), and anti-mouse IgG, HRP-linked antibody (7076S; Cell Signaling Technology, Danvers, MA, USA). Signals were detected using Chemi-Lumi One L (Nacalai Tesque) and a Multi Imager II Chemi Box (BioTools Inc., Gunma, Japan). Equal protein loading and transfer were confirmed by Ponceau S staining. Protein levels were quantified using ImageJ and normalized to the Ponceau S staining intensity.

### Fluorescent microscopic analysis

Proteasome granule formation was analyzed using an Olympus IX83 microscope (Tokyo, Japan). Ten consecutive images were acquired per field of view by stepping the focal plane along the z-axis, and proteasome condensates were counted using the entire z-stack. Proteasome condensates were then quantified using the entire z-stack. Images were processed and visually re-confirmed using ImageJ to obtain quantitative data. To ensure statistical significance, more than 100 live cells were observed per condition across three independent experiments (totaling over 300 cells).

### Measurement of intracellular pH

Intracellular pH (pHi) was measured using the genetically encoded pH sensor pHluorin2 as previously described 25, 33, 73.

### Statistical analysis

Statistical significance was evaluated using one-way ANOVA with Dunnett’s post-hoc test using RStudio (https://posit.co/products/open-source/rstudio/). Data are expressed as the mean ± standard deviation (*n* = 3).

## DATA AVAILABILITY

All data discussed in this article is available upon request

## AUTHOR CONTRIBUTIONS

S.T.: conceptualization; investigation; writing-original draft preparation. M.I.: investigation; writing-reviewing. M.H.: investigation; writing-reviewing. A.M.: investigation; writing-reviewing and editing. S.I.: conceptualization; project administration; funding acquisition; writing-reviewing and editing.

## SUPPLEMENTAL MATERIAL

All supplemental data for this article are available online at http://www.microbialcell.com/researcharticles/2026a-tanaka-microbial-cell/.

.

## CONFLICT OF INTEREST

No potential conflicts of interest were reported by the authors.

## ABBREVIATIONS

pHi – intracellular pH

PSG – proteasome storage granule

SD medium – synthetic defined medium
