# Supplementary material for: Sorbic acid triggers rapid formation of proteasome storage granules in Saccharomyces cerevisiae
Source: Microb Cell. 2026 Aug 12;13:329–39. doi: 10.15698/mic2026.08.886 (PMC13617381; doi:10.15698/mic2026.08.886)
Supplement: Supplementary file 1 [file mic-13-329-s01.pdf]

Supplemental Material

Sorbic acid triggers rapid formation of proteasome storage granules in *Saccharomyces cerevisiae*

Shoei Tanaka[1], Mitsuki Imajo[1], Mieko Hayashi[2], Akira Matsuura[2], and Shingo Izawa[1] *

[1] *Department of Applied Biology, Graduate School of Science and Technology, Kyoto Institute of Technology, Sakyo-ku, Kyoto 606-8585, Kyoto, Japan*

[2] *Department of Biology, Graduate School of Science, Chiba University, Chiba 263-8522, Japan*

Running title: Sorbic acid induces proteasome storage granule formation

* Corresponding author: Shingo Izawa Ph.D.

Tel.: +81-75-724-7764; E-mail: thioredoxin@kit.ac.jp

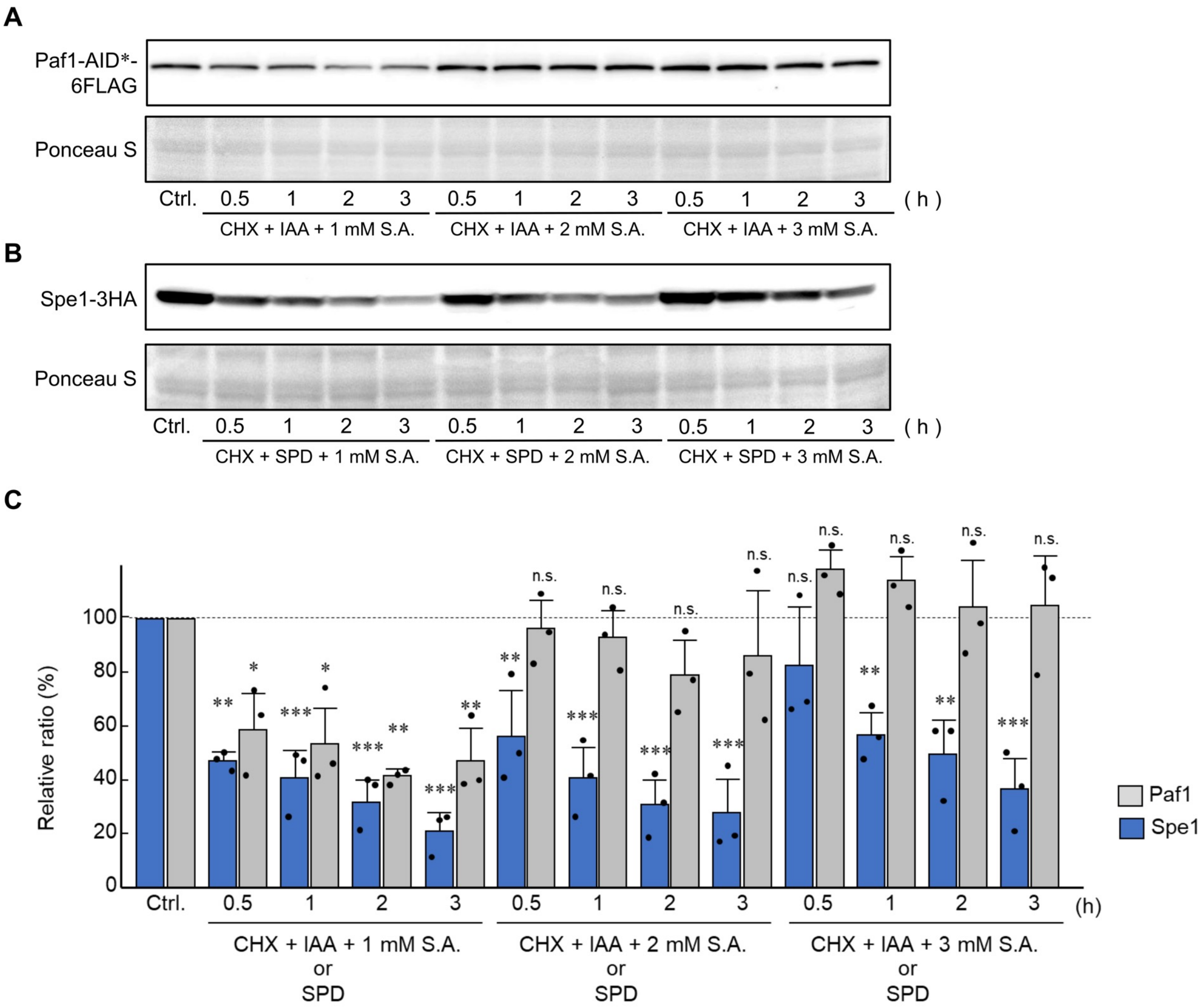

FIGURE S1. Effects of sorbic acid on proteasomal proteolysis. (**A**) Assessment of proteasomal proteolysis efficiency using the auxin-inducible degron (AID) system. Cells expressing Paf1–AID*–6FLAG were treated with 0.75 mM indole-3-acetic acid (IAA) and 200 μg/mL cycloheximide (CHX), with 1–3 mM sorbic acid, for the indicated durations. (**B**) Assessment of ubiquitin-independent proteasomal degradation using Spe1-3HA. Cells were treated with 1 mM spermidine (SPD) and 200 μg/mL CHX to induce Spe1 degradation, in the presence of 1–3 mM mM sorbic acid. (**C**) Quantitative analysis of Paf1–AID*–6FLAG and Spe1-3HA levels normalized to Ponceau S staining. Statistical significance was assessed using Dunnett's test (n = 3); **$p < 0.01$, ***$p < 0.005$. n.s., statistically not significant.
